# Survey of psychological resilience among university students majoring in long-term-care–related disciplines in Taiwan

**DOI:** 10.1186/s40359-024-02313-4

**Published:** 2024-12-26

**Authors:** Chia-Chen Chang, Chen-Yin Tung, Su-Hao Fan, Wei-Hsiang Huang

**Affiliations:** 1 Department of Health Promotion and Health Education, College of Education, National Taiwan Normal University, Taipei City, Taiwan; 2 Department of General Medicine, Cardinal Tien Hospital, New Taipei City, Taiwan; 3 Fire Department, New Taipei City Government, New Taipei City, Taiwan; 4 Department of Early Childhood Care and Education, University of Kang-Ning, Taipei City, Taiwan

**Keywords:** Long-term-care–related disciplines, University students, Psychological resilience

## Abstract

**Background:**

The psychological resilience of university students majoring in long-term-care (LTC)–related disciplines is crucial for workforce retention and effective care provision in this field. This study aims to investigate the differences in levels of psychological resilience among these students in Taiwan.

**Methods:**

This cross-sectional study involved 258 participants selected via stratified random sampling from 23 universities across Taiwan from November 2021 to November 2022, representing a diverse educational context. The research instrument used was the Resilience Scale for Adults, a validated psychological resilience questionnaire. The independent variables included participants’ demographic data, while the dependent variables encompassed five dimensions of resilience: personal strength, family cohesion, social resources, social skills, future organizational style, and total resilience score. Data analysis was performed using descriptive statistics, independent-samples t-tests, analysis of variance, and multiple regression analysis.

**Results:**

Participants with LTC work experience and leadership roles in campus clubs demonstrated significantly higher scores in personal strength (LTC: t = 2.04, *p* = 0.04, d = 0.29; leadership: t = 2.89, *p* = 0.01, d = 0.45), social resources (leadership: t = 2.47, *p* = 0.01, d = 0.34), social skills (leadership: t = 4.51, *p* = 0.01, d = 0.62), and future organizational style (LTC: t = 2.72, *p* = 0.01, d = 0.39). Higher academic performance was linked to greater personal strength, social resources, future organizational style, and total resilience (F = 4.69–3.12, *p* < 0.05). Regression analysis confirmed the predictive value of leadership experience and LTC work on various resilience dimensions. These results underscore the importance of practical exposure and extracurricular engagement in fostering resilience.

**Conclusion:**

Students engaged in club activities, leadership roles, and LTC work displayed higher psychological resilience. Educational institutions should foster club participation, leadership experiences, and partnerships with workplaces to enhance student resilience and professional readiness.

**Supplementary Information:**

The online version contains supplementary material available at 10.1186/s40359-024-02313-4.

Wei-Hsiang Huang: https://orcid.org/0000-0002-5219-7920.

## Introduction

Since 2003, Taiwan has been facing the challenges of an aging population and a severe shortage of healthcare labor [1]. The scope of long-term-care (LTC) work includes home-based care, community care, and institutional care, and the aging population creates a significant job market in this area. Research on Taiwanese healthcare workforce data from 2010 to 2019 indicates that cities near Taipei face the greatest shortages of LTC professionals, despite no increase in demand for LTC facilities [1]. This suggests that the existing LTC facilities in Taiwan lack an adequate workforce, and the proportion of graduates from relevant fields entering the LTC job market may be relatively low. Considering Taiwan’s LTC policy, it is evident that where it concerns the development and employment of LTC manpower, it promotes short career spans. Therefore, one study suggests that the government should reconsider LTC capacity and quality in its policies [2]. Further examination of the problems faced in LTC work domestically and internationally reveals that both the United States and Taiwan struggle with an inadequate workforce and a labor shortage, primarily due to aging populations and a lack of LTC services in family and community settings [3].

University students majoring in LTC-related disciplines often face unique stressors compared to students in other fields. This includes emotional challenges associated with caring for older adults, clinical internships that demand extensive physical and mental effort, and concerns about entering a demanding job market. Such challenges can exacerbate psychological distress, making psychological resilience a critical factor for their academic and professional success [4, 5].

Psychological resilience refers to an individual’s ability to withstand stress in adverse circumstances, influencing their capacity to cope with stress [6]. It can be understood as the ability to adapt positively to challenging events [7]. A study involving 141 university students demonstrated that psychological resilience significantly predicts mental health [8]. Additionally, greater psychological resilience in the workplace is linked to reduced depression, absenteeism, and productivity issues [9]. Originally emerging from psychopathology research [10], the study of resilience now spans positive psychology, adult development, and stress-coping literature.

The work performed by nursing professionals and LTC workers often involves similar stressors. A meta-analysis of data from 2009 to 2019 on newly graduated nursing professionals highlighted that work-related stress predicts resignation intentions, but higher levels of psychological resilience and team cohesion reduce these intentions [11]. This underscores the importance of resilience in retaining professionals in the demanding LTC sector, where workforce shortages remain a significant issue in Taiwan. Understanding the psychological resilience of LTC students may thus help educational institutions implement targeted reforms to enhance retention.

Research also shows that enhancing resilience among LTC workers can reduce disturbances caused by illness or changes in mental health [12]. Studies from Spain and Singapore indicate that high resilience levels reduce depression and alleviate stress-related burdens for LTC caregivers [13, 14]. Among nursing professionals, enhancing resilience has been shown to reduce burnout, improve job satisfaction, and strengthen workplace engagement [15, 16].

Previous research highlights that college students face significant stress, with increasing focus on the protective effects of resilience [17]. Students with higher resilience report better stress perception, emotional regulation, social connections, and reduced anxiety [17]. Studies involving nursing and medical students further demonstrate that resilience mediates academic stress, reduces fatigue, and improves mental health [18–20]. Thus, understanding and promoting resilience during academic years is vital for future professional success.

In summary, this study investigates differences in the psychological resilience levels among college students in Taiwan majoring in LTC-related disciplines. This research is significant due to the limited existing studies on this population and its potential to provide valuable insights for educational reforms aimed at improving student resilience.

## Methods

### Study Design and Procedure

This cross-sectional study utilized an online questionnaire to collect data between November 2021 and November 2022. Participants were selected through stratified random sampling from 23 universities across Taiwan. Invitations were sent via email, including details about the study purpose, procedures, and instructions for completing the survey. Participants who consented to participate accessed the survey through a provided link, beginning with an informed consent statement. Those selecting “Yes” proceeded to the questionnaire, while those selecting “No” exited automatically.

The survey, designed to enhance comfort and accuracy by being completed in familiar settings, took approximately 15 min. To encourage participation, respondents received a 50 TWD convenience store gift card as an incentive.

### Variable selection and measurement

The selection of variables was guided by recommendations from relevant literature.

#### Independent variables

##### Binary categorical variables

Gender, Age (below 22 years /22 years and above), LTC work experience (yes/no), non-LTC work experience (yes/no), campus club participation (yes/no), and holding a leadership role in a campus club (yes/no).

##### Ternary categorical variables

Academic performance ranking (upper, middle, or lower tertiles) and religious beliefs (Christian/Catholic, Buddhist/Taoist, and no religious belief).

#### Dependent variables

The dependent variables were measured using a validated psychological resilience questionnaire, encompassing five continuous dimensions of resilience: individual strength, family cohesion, social resources, social skills, and future organizational style. Higher scores in these dimensions indicated greater levels of psychological resilience.

**Fig. 1:**
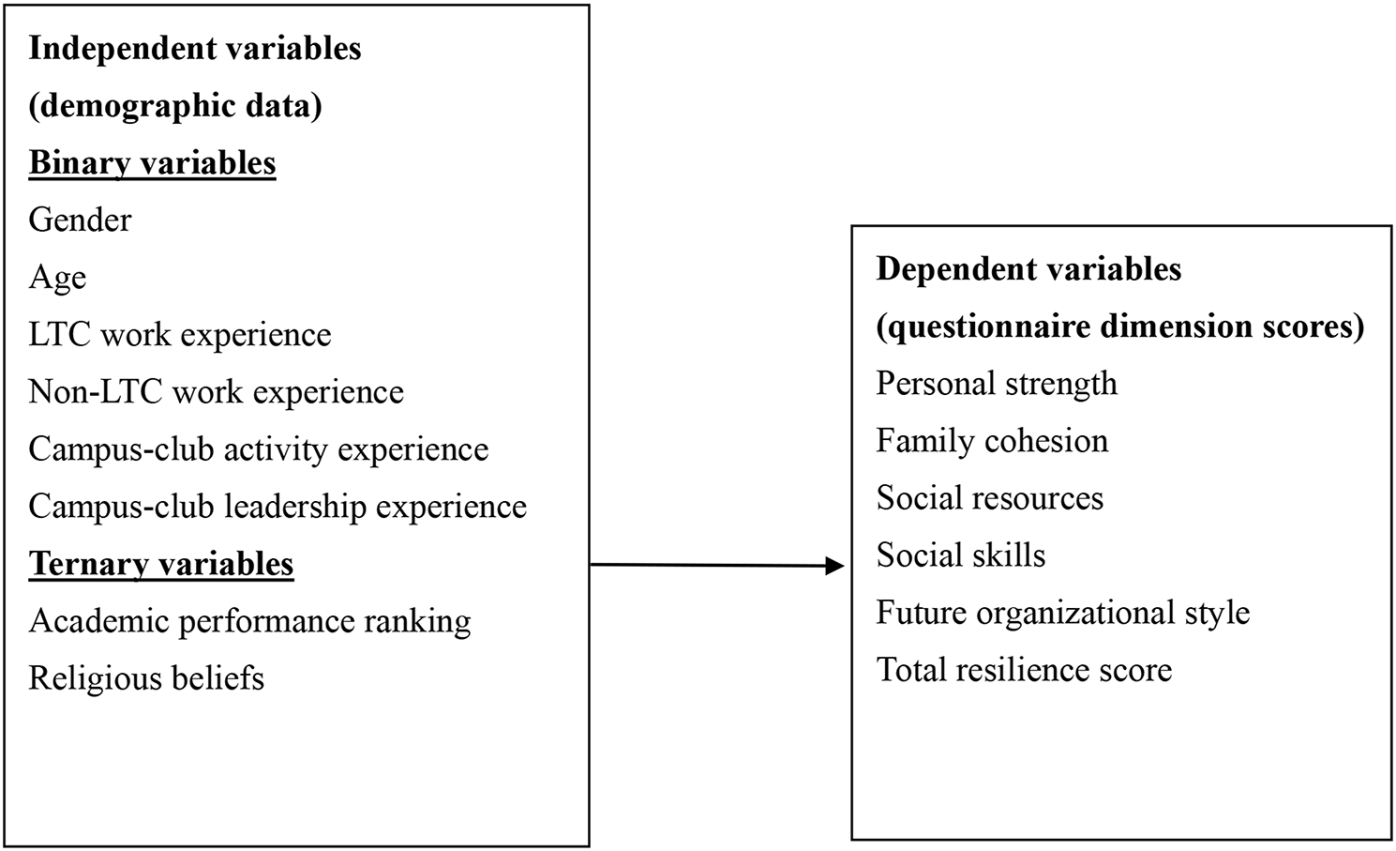
Research framework diagram. Note: Independent variables include demographic data, categorized as binary or ternary variables, while dependent variables represent the dimensions of psychological resilience

The research framework, illustrating the relationships among the independent and dependent variables, is presented in Fig. 1.

### Instrument validation

#### Measurement tool description

The Resilience Scale for Adults (RSA) was employed to assess psychological resilience in this study. Originally developed to measure resilience among burn injury patients, the RSA contains 33 items and was adapted and translated into Chinese for local application [21, 22]. The Chinese version demonstrated robust internal consistency (Cronbach’s α = 0.89) and test–retest reliability (0.89 over 3–4 weeks). Following a construct validity analysis, the scale was refined to 29 items across five dimensions: individual strength, family cohesion, social resources, social skills, and future organizational style. A seven-point Likert scale was used for responses, with higher scores reflecting greater psychological resilience.

#### Validity and reliability testing

##### Item analysis

To ensure the validity and quality of the measurement tool, item analysis was conducted using descriptive statistics, extreme-group comparisons, and homogeneity tests [23]. Items were retained if their means fell within ± 1.5 SDs of the overall mean and their SD exceeded 0.75, ensuring representativeness and variability. Items marked as reverse-scored were coded accordingly for consistency. Extreme-group comparisons retained items with a critical ratio > 3 and statistically significant differences (*p* < 0.05). Homogeneity tests ensured significant item-total correlations (*r* > 0.3) and that the Cronbach’s alpha (α) of the scale did not increase after deleting any single item. All retained items are listed in Table 1, confirming high measurement quality.

**Table 1:** Item Analysis and Reliability Assessment of the Research Instrument

Items	Descriptive statistical assessment method		Critical Ratio	Homogeneity test (total score α = 0.924)	
	Mean	SD		Correlation between items and total score (*R*)	Cronbach’s α when items are deleted
E1	4.891	1.205	6.352***	0.450	0.923
A2	4.329	1.579	7.661***	0.484	0.923
B3	5.140	1.470	7.366***	0.432	0.923
E4	4.426	1.635	10.697***	0.617	0.921
E5	4.430	1.507	9.062***	0.557	0.921
C6	5.950	1.297	9.683***	0.583	0.921
B7	5.636	1.671	9.976***	0.558	0.922
C8	4.570	1.739	8.535***	0.481	0.923
C9	5.829	1.342	11.160***	0.653	0.920
C10	5.353	1.298	9.082***	0.562	0.921
A11	5.318	1.338	9.953***	0.618	0.920
B12	5.519	1.618	7.920***	0.484	0.923
B13	5.787	1.313	10.569***	0.603	0.921
C14	5.574	1.237	9.892***	0.585	0.921
C15	5.806	1.361	10.808***	0.622	0.920
B16	5.508	1.442	11.212***	0.628	0.920
A17	4.721	1.507	12.463***	0.655	0.920
A18	4.771	1.391	9.873***	0.596	0.921
D19	4.612	1.587	12.900***	0.633	0.920
C20	5.593	1.187	9.679***	0.642	0.920
D21	4.857	1.492	11.424***	0.600	0.921
D22	5.442	1.343	11.335***	0.578	0.921
B23	5.643	1.313	9.833***	0.580	0.921
D24	4.930	1.453	11.782***	0.563	0.921
C25	5.252	1.276	10.389***	0.639	0.920
A26	4.446	1.563	9.209***	0.550	0.922
B27	4.233	1.771	5.731***	0.428	0.924
A28	5.318	1.541	10.950***	0.613	0.921
E29	5.190	1.471	8.682***	0.555	0.921

Note This table summarizes item statistics, including means, standard deviations, item-total correlations, and Cronbach’s α if deleted. Items were excluded if their mean exceeded ± 1.5 standard deviations from the scale mean (i.e., > 7.31 or < 2.97) or if their standard deviation was less than 0.75

##### Confirmatory factor analysis (CFA)

CFA was performed to assess the construct validity of the RSA, using established thresholds for model fit: RMSEA < 0.08, CFI > 0.90, and SRMR < 0.08 [24]. Convergent validity was confirmed when factor loadings exceeded 0.50, composite reliability (CR) was greater than 0.60, and the average variance extracted (AVE) surpassed 0.36 [25]. Discriminant validity was verified by ensuring that the inter-construct correlations were lower than the square root of the AVE for each construct. The CFA results demonstrated good model fit, with acceptable indices for personal strength, family cohesion, social resources, social skills, and future organizational style.

##### Reliability analysis

Internal consistency was evaluated using Cronbach’s alpha (α), with a threshold of > 0.70 indicating strong reliability [26]. The results showed high reliability across all constructs: personal strength (α = 0.78), family cohesion (α = 0.85), social resources (α = 0.84), social skills (α = 0.85), and future organizational style (α = 0.85). These findings indicate that each dimension was effectively measured, demonstrating strong consistency and homogeneity among the items.

### Participants and target population

The participants in this study were fourth-year undergraduates (or second-year students in two-year programs) from 23 colleges across Taiwan offering caregiving programs during the period from November 2021 to November 2022. To be included, participants must have completed at least 30 h of clinical internship as part of their caregiver training program. According to statistics from the Taiwan Ministry of Education in 2021, there were a total of 1,157 undergraduates enrolled in caregiving programs nationwide [27], which defined the target population for this study.

#### Sampling method

To ensure representative sampling, we employed a stratified random sampling method, following the framework used in previous nationwide college surveys [28]. Colleges were stratified by geographic region into four strata: North (9 colleges), Central (3 colleges), South (10 colleges), and East (1 college). Random selection of colleges within each stratum was conducted using Excel software’s random number generation to ensure an unbiased process. Participants from the selected colleges were further chosen using the same randomization method within Excel software, minimizing human bias. This approach maintained both regional representation and adherence to the study’s inclusion criteria concerning academic year and completion of clinical internship hours.

#### Sample size and response rate

A total of 263 questionnaires were distributed, with 258 valid and complete responses collected, resulting in a high response rate of 98.10%. To ensure sufficient statistical power for the multiple regression analysis, a sample size estimation was performed using G*Power. With a power of 0.80, an alpha level of 0.05, and an estimated effect size of 0.15 [29], a minimum sample size of 92 participants was required. Our final sample of 258 participants far exceeded this threshold, providing robust statistical power (actual power = 0.804) for detecting significant relationships among the variables of interest in the multiple regression analysis.

### Statistical analyses

Statistical analyses were conducted using IBM SPSS for Windows version 22.0, STATA version 14.0, and G*Power version 3.1.7, with a significance level of 5% for Type I error. Item analysis, confirmatory factor analysis (CFA), and reliability analysis were performed to ensure the quality and validity of the questionnaire items. Descriptive statistics, including frequency distributions, means, and standard deviations, were used to summarize participant characteristics and response distributions. Inferential statistics, such as independent-samples t-tests, analysis of variance (ANOVA), and effect sizes measured using Cohen’s d, were applied to explore relationships among variables. Multiple regression analyses were conducted to assess the impact of independent variables on psychological resilience dimensions and the total resilience score, while controlling for other variables, allowing for a more comprehensive examination of predictors and their unique contributions to outcomes.

To further investigate significant differences identified through ANOVA, planned post hoc analyses were performed using Scheffe’s test to control for potential Type I errors due to multiple comparisons. Assumptions regarding normal distribution and the independence of variables were met to ensure the robustness of the analyses.

## Results

### Demographics

The majority of participants (205, 79.5%) were female. Regarding age, 109 participants (42.3%) were below 22 years, while 149 (57.7%) were 22 years and above (Table 2). A total of 65 participants (25.2%) had experience in LTC work, whereas 193 (74.8%) did not. Non-LTC work experience was reported by 173 participants (67.1%). In terms of campus-club experience, 124 participants (48.1%) had participated in campus-club activities, and 74 (28.7%) held leadership roles in campus clubs. For academic performance, 109 students (42.3%) ranked in the upper tertile, 119 (46.1%) in the middle tertile, and 30 (11.6%) in the lower tertile. Most participants (187, 72.5%) identified with Buddhist or Taoist beliefs.

**Table 2:** Demographic characteristics of participants (*N* = 258)

Demographic variable	Group	Number (*N*)	Percentage (%)
Gender	Male	53	20.5%
	Female	205	79.5%
Age	Under 22	109	42.3%
	Over 22	149	57.7%
LTC work experience	Y	65	25.2%
	N	193	74.8%
Non-LTC work experience	Y	173	67.1%
	N	85	32.9%
Campus-club activities	Y	124	48.1%
	N	134	51.9%
Campus-club leadership	Y	74	28.7%
	N	184	71.3%
Academic ranking (tertiles)	Upper	109	42.2%
	Middle	119	46.1%
	Lower	30	10.5%
Religious beliefs	Catholicism and Christianity	35	13.6%
	Buddhism and Taoism	187	72.5%
	No beliefs	36	14.0%

### Responses for each dimension and overall resilience

For the personal strength construct, which comprised six items, the average score was 28.90, with a SD of 6.18 (Table 3). The highest-scoring items were A11 (“My personal issues”) and A28 (“When things get tough, I tend to”), with average scores of 5.32 (SDs of 1.34 and 1.54, respectively). The lowest-scoring item was A2 (“When unexpected things happen”), with an average score of 4.33 (SD = 1.58). For family cohesion, the average score was 37.47 (SD = 7.44), with B13 (“My family characteristics are”) scoring the highest (mean = 5.79, SD = 1.31) and B27 (“In my family, family members like to”) scoring the lowest (mean = 4.23, SD = 1.77).

**Table 3:** Summary of items and response statistics for psychological resilience constructs (*N* = 258)

Construct	Item name	Number of items	Total mean	Total SD	Item mean	Item SD
Personal strength	#A2. When unexpected things happen …	6	28.90	6.18	4.33	1.58
	A11. My personal issues				5.32	1.34
	#A17. My abilities				4.72	1.51
	A18. My judgment and decisions				4.77	1.39
	#A26. I’m skilled at				4.45	1.56
	A28. When things get tough, I tend to				5.32	1.54
Family cohesion	B3. The experiences in my family about what is important in life	7	37.47	7.44	5.14	1.47
	#B7. I feel				5.64	1.67
	#B12. When family members experience a crisis or emergency				5.52	1.62
	B13. My family characteristics are				5.79	1.31
	#B16. When things get tough, my family				5.51	1.44
	B23. Facing others, my family members show				5.64	1.31
	B27. In my family, family members like to				4.23	1.77
Social resources	C6. I can discuss personal matters with someone	8	43.93	7.46	5.95	1.30
	#C8. I enjoy				4.57	1.74
	#C9. Those who are good at encouraging me are				5.83	1.34
	C10. The connections I have with my friends are				5.35	1.30
	C14. Staying flexible in social situations				5.57	1.24
	#C15. The support I receive comes from				5.81	1.36
	C20. When needed				5.59	1.19
	#C25. My close friends or family members				5.25	1.28
Social skills	#D19. New friendships	4	19.84	4.86	4.61	1.59
	D21. Making new friends				4.86	1.49
	#D22. When I’m with others				5.44	1.34
	D24. Coming up with a good topic for conversation is				4.93	1.45
Future organizational style	E1. My future plans are	4	18.94	4.85	4.89	1.20
	#E4. I feel my future is				4.43	1.64
	#E5. How to achieve my future goals				4.43	1.51
	E29. My future goals are clear				5.19	1.47
					**minimum**	**maximum**
**Total psychological resilience score**		29	149.07	23.83	88	201

Note: This table summarizes descriptive statistics for psychological resilience constructs, including means and standard deviations. Items marked with “# reverse-scored” are reverse-coded for consistent score interpretation, with higher scores indicating stronger resilience

In the social resources construct, the average score was 43.93 (SD = 7.46), with C6 (“I can discuss personal matters with someone”) scoring the highest (mean = 5.95, SD = 1.30) and C8 (“I enjoy”) scoring the lowest (mean = 4.57, SD = 1.74). Social skills had an average score of 19.84 (SD = 4.86), with D22 (“When I’m with others”) scoring the highest (mean = 5.44, SD = 1.34) and D19 (“New friendships”) scoring the lowest (mean = 4.61, SD = 1.59). For future organizational style, the average score was 18.94 (SD = 4.85), with E29 (“My future goals are clear”) scoring the highest (mean = 5.19, SD = 1.47). The lowest-scoring items were E4 (“I feel my future is”) and E5 (“How to achieve my future goals”), each with an average score of 4.43 (SDs = 1.64 and 1.51, respectively). The overall psychological resilience score, encompassing all 29 items, had an average score of 149.07 (SD = 23.83), with a range of 88 to 201.

### Independent and dependent variable relationships: analysis and findings

#### Bivariate analysis results for psychological resilience dimensions

(1) The initial analyses involved independent-samples t-tests and ANOVA to assess differences in psychological resilience dimensions based on various demographic factors. The key findings are summarized as follows:

- Independent-samples t-Test results (table 4)
  **Personal strength**: Participants with LTC work experience scored higher (M = 30.25, SD = 6.53) compared to those without (M = 28.45, SD = 6.01), t = 2.04, *p* = 0.04, d = 0.29. Similarly, participants with non-LTC work experience scored higher (M = 29.49, SD = 6.40) than those without (M = 27.72, SD = 5.58), t = 2.28, *p* = 0.02, d = 0.29. Campus club experience and leadership roles were also associated with higher scores, with leadership roles having the largest effect size (M = 30.64, SD = 6.21 vs. M = 28.20, SD = 6.05, t = 2.89, *p* = 0.01, d = 0.40).
  **Social resources**: Campus club leaders scored higher (M = 45.72, SD = 7.42) than non-leaders (M = 43.21, SD = 7.37), t = 2.47, *p* = 0.01, d = 0.34.
  **Social skills**: Campus club participants scored higher (M = 20.82, SD = 4.72) compared to non-participants (M = 18.94, SD = 4.84), t = 3.15, *p* = 0.01, d = 0.39. Leadership roles further increased scores (M = 21.92, SD = 4.26 vs. M = 19.00, SD = 4.85), t = 4.51, *p* = 0.01, d = 0.62.
  **Future organizational style**: Participants with LTC work experience scored higher (M = 20.33, SD = 5.08) than those without (M = 18.47, SD = 4.70), t = 2.72, *p* = 0.01, d = 0.39.
- ANOVA results (table 5)
  **Personal strength**: A significant difference was observed based on academic performance (F = 4.69, *p* = 0.01, η² = 0.04). Scheffe’s post hoc analysis indicated that high-performing students (M = 30.05, SD = 6.33) scored significantly higher than low-performing students (M = 26.26, SD = 6.08).
  **Social resources**: Significant differences were found based on academic performance (F = 3.12, *p* = 0.04, η² = 0.03). Scheffe’s post hoc test revealed that high-performing students (M = 44.59, SD = 6.75) scored higher than low-performing students (M = 40.63, SD = 9.56).
  **Future organizational style**: Differences based on academic performance were significant (F = 3.68, *p* = 0.03, η² = 0.03). Scheffe’s post hoc analysis showed that high-performing students (M = 19.40, SD = 5.04) had higher scores than low-performing students (M = 16.63, SD = 5.17).
  **Family cohesion (religious beliefs)**: Significant differences were observed (F = 3.74, *p* = 0.03, η² = 0.03). Scheffe’s post hoc analysis indicated that participants identifying with Buddhist/Taoist beliefs (M = 38.10, SD = 6.55) scored higher than those with no religious affiliation (M = 34.44, SD = 8.83).
- Total psychological resilience score
  **Independent-Samples t-Test (**Table 4**)**: Campus club participants had higher scores (M = 153.28, SD = 24.01) compared to non-participants (M = 145.18, SD = 23.06), t = 2.76, *p* = 0.01, d = 0.34. Leadership roles were associated with even higher scores (M = 156.64, SD = 24.41 vs. M = 146.03, SD = 22.96), t = 3.29, *p* = 0.01, d = 0.45.
  **ANOVA (**Table 5**)**: Significant differences were found based on academic performance (F = 3.33, *p* = 0.04, η² = 0.03), with high performers scoring higher (M = 151.86, SD = 23.39) than low performers (M = 138.78, SD = 23.69).

**Table 4:** Results of independent-samples t-Test for psychological resilience dimensions (*N* = 258)

**Variable**	**Items**	***N***	**Personal strength**					**Family cohesion**					**Social resources**					
			**Mean**	**SD**	**t**	***p***	**d**	**Mean**	**SD**	**t**	***p***	**d**	**Mean**	**SD**	**t**		***p***	**d**
**Gender**	Male	53	27.96	6.94	−1.24	0.22	0.19	36.15	7.63	−1.44	0.15	0.22	42.06	8.40	−1.87		0.07	0.32
	Female	205	29.15	5.97				37.81	7.37				44.41	7.14				
**Age**	Under 22	109	28.41	5.61	−1.12	0.27	0.14	37.98	7.20	0.95	0.34	0.12	43.73	7.60	−0.35		0.72	0.05
	Over 22	149	29.26	6.57				37.09	7.61				44.07	7.38				
LTC work experience	Y	65	30.25	6.53	2.04	0.04	0.29	37.52	8.45	0.07	0.94	0.01	44.72	7.65	1.00		0.32	0.14
	N	193	28.45	6.01				37.45	7.09				43.66	7.40				
Non-LTC work experience	Y	173	29.49	6.40	2.28	0.02	0.29	37.54	7.47	0.22	0.82	0.03	44.39	7.50	1.44		0.15	0.19
	N	85	27.72	5.58				37.32	7.42				42.98	7.34				
Campus-club activities	Y	124	29.85	5.80	2.38	0.02	0.30	38.23	7.91	1.58	0.11	0.16	44.81	7.47	1.83		0.07	0.23
	N	134	28.03	6.42				36.76	6.94				43.11	7.39				
Campus-club leadership	Y	74	30.64	6.21	2.89	0.01	0.40	38.66	7.68	1.64	0.10	0.23	45.72	7.42	2.47		0.01	0.34
	N	184	28.20	6.05				36.98	7.31				43.21	7.37				
**Variable**	**Items**	**N**	**Social skills**					**Future organizational style**					**Total psychological resilience score**					
			**Mean**	**SD**	***t***	**p**	***d***	**Mean**	**SD**	***t***	**p**	***d***	**Mean**	**SD**		***t***	**p**	***d***
**Gender**	Male	53	19.43	4.92	−0.68	0.50	0.11	18.87	5.45	−0.12	0.91	0.02	144.47	25.40		−1.58	0.12	0.24
	Female	205	19.95	4.86				18.96	4.70				150.26	23.32				
**Age**	Under 22	109	19.77	4.94	−0.20	0.84	0.03	18.31	4.09	−1.85	0.07	0.23	148.21	22.16		−0.50	0.62	0.06
	Over 22	149	19.89	4.82				19.40	5.32				149.70	25.03				
LTC work experience	Y	65	20.85	4.55	1.94	0.05	0.28	20.33	5.08	2.72	0.01	0.39	153.68	24.80		1.81	0.07	0.26
	N	193	19.50	4.93				18.47	4.70				147.52	23.35				
Non-LTC work experience	Y	173	20.16	4.94	1.49	0.14	0.20	19.50	4.91	2.70	0.01	0.27	151.08	24.14		1.94	0.05	0.26
	N	85	19.20	4.67				17.79	4.57				145.00	22.77				
Campus-club activities	Y	124	20.82	4.72	3.15	0.01	0.39	19.59	4.39	2.08	0.04	0.26	153.28	24.01		2.76	0.01	0.34
	N	134	18.94	4.84				18.34	5.20				145.18	23.06				
Campus-club leadership	Y	74	21.92	4.26	4.51	0.01	0.62	19.70	4.54	1.61	0.11	0.22	156.64	24.41		3.29	0.01	0.45
	N	184	19.00	4.85				18.63	4.96				146.03	22.96				

Note This table presents the results of independent-samples t-tests comparing psychological resilience dimensions across groups based on demographic variables. Effect sizes (Cohen’s d) are reported and interpreted as small (0.2), medium (0.5), and large (0.8)

**Table 5:** Results of ANOVA for psychological resilience dimensions and total score (*N* = 258)

**Variable**	**Items**	***N***	**Personal strength**						**Family cohesion**					
			**Mean**	**SD**	**F**	***p***	**η** ^**2**^	**Scheffe**	**Mean**	**SD**	**F**	***P***	**η** ^**2**^	**Scheffe**
Academic ranking	Upper	109	30.05	6.33	4.69	0.01	0.04	Upper > middle	37.70	7.53	0.45	0.64	0.04	
	Middle	119	28.50	5.91					37.61	7.21				
	Lower	30	26.26	6.08					36.22	8.22				
Religious beliefs	Catholicism & Christianity	35	29.43	6.82	0.60	0.55	0.01		37.20	9.59	3.74	0.03	0.03	Buddhism & Taoism > No beliefs
	Buddhism & Taoism	187	29.00	6.16					38.10	6.55				
	No beliefs	36	27.92	5.73					34.44	8.83				
**Variable**	**Items**	**N**	**Social resources**						**Social skills**					
			**Mean**	**SD**	***F***	**p**	**η** ^**2**^	**Scheffe**	**Mean**	**SD**	***F***	**p**	**η** ^**2**^	**Scheffe**
Academic ranking	Upper	109	44.59	6.75	3.12	0.04	0.03	Upper > Lower	20.13	4.78	0.56	0.57	0.04	
	Middle	119	44.19	7.41					19.77	4.71				
	Lower	30	40.63	9.56					19.04	6.01				
Religious beliefs	Catholicism & Christianity	35	43.37	9.08	0.41	0.67	0.01		19.83	5.01	1.26	0.29	0.01	
	Buddhism & Taoism	187	44.18	7.35					20.07	4.97				
	No beliefs	36	43.14	6.33					18.67	4.06				
**Variable**	**Items**	**N**	**Future organizational style**						**Total psychological resilience score**					
			**Mean**	**SD**	***F***	**p**	**η** ^**2**^	**Scheffe**	**Mean**	**SD**	***F***	**p**	**η** ^**2**^	**Scheffe**
Academic ranking	Upper	109	19.40	5.04	3.68	0.03	0.03	Upper > Lower	151.86	23.39	3.33	0.04	0.03	Upper > Lower
	Middle	119	19.08	4.48					149.15	23.78				
	Lower	30	16.63	5.17					138.78	23.69				
Religious beliefs	Catholicism & Christianity	35	20.26	5.08	1.51	0.22	0.01		150.09	29.27	1.45	0.24	0.01	
	Buddhism & Taoism	187	18.75	4.90					150.09	23.65				
	No beliefs	36	18.64	4.30					142.81	17.82				

Note This table presents ANOVA results for psychological resilience dimensions and total score across groups. Post-hoc Scheffé tests identify significant differences between specific groups. Effect sizes (η²) are reported and interpreted as small (0.01), medium (0.06), and large (0.14)

#### Multiple regression analysis results

To further investigate the relationships identified in the bivariate analyses, variables with significant differences were transformed into dummy variables and included in multiple regression models to control for potential confounders. This section reports only the significant predictors (*p* < 0.05), as summarized in Table 6. For transparency, the complete regression model, which includes both significant and non-significant predictors, is provided in Appendix 1.

**Table 6:** Significant predictors in multiple regression analysis for psychological resilience dimensions and total score (*N* = 258)

Variable	B	SE	β	t	*p*	*R* ^2^	Adj *R*^2^
**Family cohesion**						0.03	0.02
**Intercept**	37.96	0.49		76.88	< 0.001		
Religious beliefs (Ref: No beliefs)	-0.35	1.32	-0.16	-2.66	0.01		
**Social resources**						0.05	0.04
**Intercept**	43.68	0.57		76.76	< 0.001		
Campus-club leadership (Ref: N)	2.38	1.02	0.14	2.34	0.02		
Academic ranking (Ref: lower)	-3.58	1.49	-0.15	-2.40	0.02		
**Social skills**						0.07	0.07
**Intercept**	18.94	0.41		46.66	< 0.001		
Campus-club leadership (Ref: N)	2.74	0.86	0.26	3.18	0.01		
**Future organizational style**						0.10	0.09
**Intercept**	17.06	0.61		28.11	< 0.001		
LTC work experience (Ref: N)	1.98	0.68	0.18	2.91	0.01		
Non-LTC work experience (Ref: N)	1.47	0.62	0.14	2.35	0.02		
Campus-club activities (Ref: N)	1.44	0.59	0.15	2.45	0.02		
Academic ranking (Ref: lower)	-2.60	0.95	-0.17	-2.75	0.01		
**Total psychological resilience score**						0.07	0.05
**Intercept**	146.54	2.09		70.13	< 0.001		
Campus-club leadership (Ref: N)	8.33	4.31	0.16	1.93	0.05		
Academic ranking (Ref: lower)	-10.72	4.73	-0.14	-2.27	0.02		

Note: This table summarizes only the significant predictors (*p* < 0.05) identified in the multiple regression analysis. The full regression model, including both significant and non-significant predictors, is provided in Appendix 1 for transparency

#### Regression analysis results (table 6)

- **Family cohesion**: Religious beliefs significantly predicted family cohesion (B = -0.35, *p* = 0.01), with individuals identifying with a religion scoring lower than those without any religious affiliation. This model accounted for 3% of the variance (R² = 0.03, adjusted R² = 0.02).
- **Social resources**: Campus club leadership was positively associated with social resources (B = 2.38, *p* = 0.02), while lower academic performance showed a negative association (B = -3.58, *p* = 0.02). This model explained 5% of the variance (R² = 0.05, adjusted R² = 0.04).
- **Social skills**: Campus club leadership emerged as a significant positive predictor of social skills (B = 2.74, *p* = 0.01). The model explained 7% of the variance (R² = 0.07, adjusted R² = 0.07).
- **Future organizational style**: Positive associations were observed for long-term care work experience (B = 1.98, *p* = 0.01), non-long-term care work experience (B = 1.47, *p* = 0.02), and campus club participation (B = 1.44, *p* = 0.02), while lower academic performance negatively predicted future organizational style (B = -2.60, *p* = 0.01). This model accounted for 10% of the variance (R² = 0.10, adjusted R² = 0.09).
- **Total resilience score**: Campus club leadership showed a marginally significant positive association with the total psychological resilience score (B = 8.33, *p* = 0.05), while lower academic performance was significantly negatively associated (B = -10.72, *p* = 0.02). This model explained 7% of the variance (R² = 0.07, adjusted R² = 0.05).

## Discussion

This study primarily investigated the psychological resilience of university students in Taiwan majoring in LTC-related disciplines. Prior research has indicated that work-related stress in nursing can predict intention to resign. However, individuals with higher levels of psychological resilience and team cohesion are less likely to develop such intentions [11]. Therefore, we explored patterns in psychological resilience among university students in LTC-related disciplines based on various demographic variables. Our findings provide valuable insights for educational institutions seeking to implement reforms that could enhance workforce retention.

Our analysis of binary categorical demographic data revealed that students with LTC work experience, non-LTC work experience, extracurricular activity participation, and leadership roles in such activities scored significantly higher on measures of personal strength, social resources, social skills, future organizational style, and overall psychological resilience. This suggests that practical experience and extracurricular involvement may play a role in building resilience.

These findings align with prior research. For example, a study involving 498 nursing professionals found that individuals with less work experience exhibited higher psychological resilience compared to those with over five years of experience [30]. Additionally, a study of 178 adolescent students highlighted a significant positive association between happiness, campus activity participation, and psychological resilience [31]. Similarly, research involving 945 high school students indicated that school activity participation can enhance psychological resilience and subjective wellbeing [32].

Further analysis of ternary categorical data revealed that students with better academic performance scored significantly higher in personal strength, social resources, future organizational style, and overall psychological resilience. Religious beliefs were also significantly associated with family cohesion. This aligns with a study conducted in Japan involving 229 nursing students, which demonstrated a significant link between psychological resilience and academic performance [33]. Another study involving 118 medical students reported a similar association between psychological resilience and academic achievement [34]. Moreover, a meta-analysis of 34 studies found a moderate positive correlation between religious beliefs and psychological resilience [35].

Age was positively correlated with personal strength, social skills, and future organizational style, indicating that these aspects of psychological resilience tend to increase with age. This finding is consistent with a study showing that nursing professionals over the age of 40 exhibit higher levels of psychological resilience than younger nurses [30].

Lastly, our results showed no statistically significant differences in psychological resilience between genders. The gender distribution in this study (20.5% male, 79.5% female) was similar to a prior study on psychological resilience among medical students (21.2% male, 78.8% female [34]), which also reported no gender-based differences. This similarity in gender distribution may account for the congruence in findings.

### Limitations of the study

#### Internal validity and measurement bias

This study utilized a cross-sectional survey method, which identifies associations between variables but cannot establish causal relationships. Future research should consider experimental or longitudinal designs to address this limitation. Additionally, the use of an online questionnaire, necessitated by the COVID-19 pandemic, may have introduced measurement bias. The remote nature of data collection posed challenges in ensuring respondents fully understood the questions, and the self-reported measures are susceptible to social desirability bias, potentially affecting data accuracy.

#### External validity and generalizability

The study sample consisted exclusively of university students majoring in LTC-related disciplines, which restricts the generalizability of the findings to broader populations, such as practicing LTC professionals. This specificity may impact the external validity and application of the results to other contexts, necessitating caution when interpreting their relevance.

### Recommendations for future research and practice

#### Recommendations for future research

While this study utilized a survey to achieve its objectives, it is crucial to acknowledge that this approach can only reveal associations between variables and cannot establish causal relationships. We recommend that future researchers consider employing experimental research methods to explore and confirm causal relationships between variables. Additionally, this study’s post hoc analyses, while informative, carry a potential risk of Type I errors due to multiple comparisons. We suggest that future research incorporate larger sample sizes and employ more stringent multiple comparison correction methods to further mitigate this risk and enhance the robustness of findings.

#### Practical recommendations for educational institutions

Our findings indicate that university students with experience as club leaders scored higher for personal strength (t = 2.89, *p* = 0.01, d = 0.40), social resources (t = 2.47, *p* = 0.01, d = 0.34), and social skills (t = 4.51, *p* = 0.01, d = 0.62) than those without such experience. This pattern was further supported by multiple regression analysis, which revealed that campus club leadership remained a significant positive predictor of social resources (B = 2.38, *p* = 0.02) and social skills (B = 2.74, *p* = 0.01). Therefore, educational institutions should encourage students to participate actively in extracurricular activities and seek leadership roles in these settings.

Moreover, students with work experience in LTC-related fields demonstrated higher levels of personal strength (t = 2.04, *p* = 0.04, d = 0.29) and future organizational style (t = 2.72, *p* = 0.01, d = 0.39) compared to their peers without such experience. Regression results similarly highlighted the positive association between LTC work experience and future organizational style (B = 1.98, *p* = 0.01). Educational institutions should consider strengthening collaborations with relevant workplace partners to expand internship opportunities, which may bolster students’ psychological resilience and enhance their readiness for professional roles.

## Conclusions

This study revealed that LTC work experience, non-LTC work experience, participation in extracurricular activities, and leadership roles were linked to higher levels of psychological resilience, including personal strength, social resources, social skills, and future organizational style. Multiple regression analysis confirmed that campus club leadership and strong academic performance were significant predictors of resilience. Students involved in extracurricular activities, especially in leadership roles, exhibited greater overall resilience. Additionally, LTC work experience was associated with higher personal strength and future organizational style, highlighting the value of practical exposure in developing resilience-related skills.

### Implications for practice, education, and workforce development

This study addresses the challenges of recruiting graduates from long-term care (LTC)-related disciplines into Taiwan’s LTC workforce by examining factors that influence students’ psychological resilience, which is crucial for reducing workplace mental health issues and increasing professional retention [9].

Our findings highlight that practical LTC work experience, active campus engagement, and strong academic performance significantly enhance students’ resilience. Involvement in campus clubs, especially in leadership roles, and higher academic achievement were linked to improved personal strength, social resources, and social skills. Similarly, LTC-related work experience was associated with stronger personal strength and a future-oriented organizational style.

To better prepare students for caregiving roles, educational institutions should prioritize hands-on learning, extracurricular involvement, leadership development, and partnerships with relevant workplaces to expand internships. These efforts will enhance resilience, address LTC workforce shortages, and support mental health in healthcare settings.

## Electronic supplementary material

Below is the link to the electronic supplementary material.

Supplementary Material 1

## Data Availability

The datasets generated and/or analyzed during the current study are not publicly available because of the regulations stipulated by the Institutional Review Board of the National Taiwan Normal University’s Research Ethics Review Committee. However, the datasets are available from the first author upon reasonable request.
