# Supplementary material for: Survey of psychological resilience among university students majoring in long-term-care–related disciplines in Taiwan
Source: BMC Psychol. 2024 Dec 26;12:786. doi: 10.1186/s40359-024-02313-4 (PMC11674181; doi:10.1186/s40359-024-02313-4)
Supplement: Supplementary file 1 — Supplementary Material 1 [file 40359_2024_2313_MOESM1_ESM.docx]

**Appendix 1.** Complete Results of Multiple Regression Analysis for Psychological Resilience Dimensions and Total Score (N = 258)

| **Variable** | **B** | **SE** | **β** | **t** | **p** | **R^2^** | **Adj R^2^** |
| --- | --- | --- | --- | --- | --- | --- | --- |
| **Personal strength** |  |  |  |  |  | 0.07 | 0.05 |
| **Intercept** | 26.87 | 0.87 |  | 30.77 | <0.001 |  |  |
| LTC work experience (Ref: N) | 1.72 | 0.90 | 0.12 | 1.92 | 0.06 |  |  |
| Non-LTC work experience (Ref: N) | 1.36 | 0.83 | 0.10 | 1.64 | 0.10 |  |  |
| Campus-club activities (Ref: N) | 1.23 | 1.05 | 0.10 | 1.18 | 0.24 |  |  |
| Campus-club leadership (Ref: N) | 1.36 | 1.15 | 0.10 | 1.18 | 0.24 |  |  |
| Academic ranking (Ref: middle) | -0.58 | 0.76 | -0.05 | -0.76 | 0.45 |  |  |
| **Family cohesion** |  |  |  |  |  | 0.03 | 0.02 |
| **Intercept** | 37.96 | 0.49 |  | 76.88 | <0.001 |  |  |
| Religious beliefs (Ref: No beliefs) | -0.35 | 1.32 | -0.16 | -2.66 | 0.01 |  |  |
| **Social resources** |  |  |  |  |  | 0.05 | 0.04 |
| **Intercept** | 43.68 | 0.57 |  | 76.76 | <0.001 |  |  |
| Campus-club leadership (Ref: N) | 2.38 | 1.02 | 0.14 | 2.34 | 0.02 |  |  |
| Academic ranking (Ref: lower) | -3.58 | 1.49 | -0.15 | -2.40 | 0.02 |  |  |
| **Social skills** |  |  |  |  |  | 0.07 | 0.07 |
| **Intercept** | 18.94 | 0.41 |  | 46.66 | <0.001 |  |  |
| Campus-club activities (Ref: N) | 0.24 | 0.78 | 0.03 | 0.31 | 0.76 |  |  |
| Campus-club leadership (Ref: N) | 2.74 | 0.86 | 0.26 | 3.18 | 0.01 |  |  |
| **Future organizational style** |  |  |  |  |  | 0.10 | 0.09 |
| **Intercept** | 17.06 | 0.61 |  | 28.11 | <0.001 |  |  |
| LTC work experience (Ref: N) | 1.98 | 0.68 | 0.18 | 2.91 | 0.01 |  |  |
| Non-LTC work experience (Ref: N) | 1.47 | 0.62 | 0.14 | 2.35 | 0.02 |  |  |
| Campus-club activities (Ref: N) | 1.44 | 0.59 | 0.15 | 2.45 | 0.02 |  |  |
| Academic ranking (Ref: lower) | -2.60 | 0.95 | -0.17 | -2.75 | 0.01 |  |  |
| **Total psychological resilience score** |  |  |  |  |  | 0.07 | 0.05 |
| **Intercept** | 146.54 | 2.09 |  | 70.13 | <0.001 |  |  |
| Campus-club activities (Ref: N) | 3.00 | 3.90 | 0.06 | 0.77 | 0.44 |  |  |
| Campus-club leadership (Ref: N) | 8.33 | 4.31 | 0.16 | 1.93 | 0.05 |  |  |
| Academic ranking (Ref: lower) | -10.72 | 4.73 | -0.14 | -2.27 | 0.02 |  |  |

Note: This appendix presents the complete regression model, including significant and non-significant predictors, for a comprehensive understanding of the analysis. Significant predictors summarized in Table 6 are highlighted here for consistency.
